# Validating silicon polytrodes with paired juxtacellular recordings: method and dataset

**DOI:** 10.1152/jn.00103.2016

**Published:** 2016-06-15

**Authors:** Joana P. Neto, Gonçalo Lopes, João Frazão, Joana Nogueira, Pedro Lacerda, Pedro Baião, Arno Aarts, Alexandru Andrei, Silke Musa, Elvira Fortunato, Pedro Barquinha, Adam R. Kampff

**Affiliations:** ^1^Champalimaud Neuroscience Programme, Champalimaud Centre for the Unknown, Lisbon, Portugal;; ^2^Departamento de Ciência dos Materiais, CENIMAT/I3N and CEMOP/Uninova, Caparica, Portugal;; ^3^IMEC, Leuven, Belgium;; ^4^ATLAS Neuroengineering; and; ^5^Sainsbury Wellcome Centre, University College London, London, United Kingdom

**Keywords:** extracellular action potential, spike sorting, ground truth, juxtacellular recording, polytrodes

## Abstract

Cross-validating new methods for recording neural activity is necessary to accurately interpret and compare the signals they measure. Here we describe a procedure for precisely aligning two probes for in vivo “paired-recordings” such that the spiking activity of a single neuron is monitored with both a dense extracellular silicon polytrode and a juxtacellular micropipette. Our new method allows for efficient, reliable, and automated guidance of both probes to the same neural structure with micrometer resolution. We also describe a new dataset of paired-recordings, which is available online. We propose that our novel targeting system, and ever expanding cross-validation dataset, will be vital to the development of new algorithms for automatically detecting/sorting single-units, characterizing new electrode materials/designs, and resolving nagging questions regarding the origin and nature of extracellular neural signals.

## Abstract (precis)

*Recording in vivo from the same neuron with two different methods is difficult. It requires blindly moving each probe to within ∼100 μm of one another and for this reason such “dual-recordings” are rare. However, comparing the signals measured by different techniques is necessary to understand what they measure. We developed a method to precisely align the axes of two manipulators and used it to gather a “ground truth” dataset for dense extracellular polytrodes*.

## NEW & NOTEWORTHY

*Recording in vivo from the same neuron with two different methods is difficult. It requires blindly moving each probe to within ∼100 μm of one another and for this reason such “dual-recordings” are rare. However, comparing the signals measured by different techniques is necessary to understand what they measure. We developed a method to precisely align the axes of two manipulators and used it to gather a “ground truth” dataset for dense extracellular polytrodes*.

understanding how the brain works will require tools capable of measuring neural activity at a network scale, i.e., recording from thousands of individual neurons (Buzsáki 2004). Technical advances have driven progress in large-scale neural recordings, and the development of microfabricated silicon polytrodes has led to an exponential increase in the number of neurons that can be simultaneously monitored (Stevenson and Kording 2011; Berényi et al. 2014; Michon et al. 2014). However, each improvement in recording technology inevitably raises new questions about the nature of the signal and demands new analysis methods to interpret these growing datasets.

Extracellular recording is unique in its ability to record populations of neurons deep in the brain with submillisecond resolution; it also poses particularly daunting challenges for analysis. Each electrode is sensitive to the spiking activity of hundreds of neurons in its vicinity, and sorting this cacophony into individual sources is a challenge (Marblestone et al. 2013). Furthermore, fundamental questions regarding how each neuron participates in the bulk extracellular signal remain unresolved: How many neurons contribute to the signal detected by an electrode? How does a neuron's contribution decay with distance from the probe? Do different types of neurons have different extracellular signatures? Are extracellular recordings biased for particular types of neurons? How does the presence of the probe interfere with the activity of the surrounding neural tissue? Answers to these questions will require experiments to validate existing and future extracellular electrode technology as well as new analysis methods to interpret their data.

Employing modern methods for integrated circuit design and fabrication, probes with thousands, or even millions, of discrete sites are now being developed (Dombovári et al. 2014; Ruther and Paul 2015; Shobe et al. 2015). These devices will densely sample the extracellular electric field, such that one nearby neuron will be detected by many individual electrodes, and will thus provide a detailed description of the spatiotemporal profile of a neuron's extracellular action potential (EAP). It is expected that this additional detail will significantly aid analysis methods for the detection and isolation, and possibly type identification, of individual neurons in the vicinity of the probe, yet methods capable of utilizing such a dense sampling are just now being developed (Rossant et al. 2016).

“Ground truth” data, for which one knows exactly when a neuron in the vicinity of an extracellular probe generates an action potential, are necessary to validate the performance of these new recording devices and analysis procedures. However, the validation datasets currently available for extracellular recordings only exist for tetrodes and single-wire electrodes (Wehr et al.1999; Henze et al. 2000; Chorev and Brecht 2012; Henze and Buzsáki 2007) or are from in vitro preparations (e.g., slices; Anastassiou et al. 2015) in which the majority of background neural activity has been surgically removed. Evaluating the existing silicon polytrodes, as well as forthcoming ultra-high density CMOS probes, in vivo will require new datasets, and, ideally, new methods for efficiently gathering this vital cross-validation data.

Targeting a single neuron close to an extracellular probe with another electrode requires accurately positioning both devices deep in neural tissue. When performed blindly, the efficiency of achieving paired-recordings in which one neuron is detected by both probes is rather low, making such validation experiments much more difficult than just haphazardly recording extracellular neural signals. For this reason, such datasets are very rare; however, the ones that do exist (for tetrodes in the hippocampus) have been invaluable (Harris et al. 2000; Gold et al. 2007). We anticipate that a large amount of such validation data will be required to characterize the large-scale neural recording devices currently being developed, and we thus set out to make paired-recordings easier.

In the following we report a new method for efficiently and reliably targeting, blindly, two different recording devices to the same region in the brain. This method was then used to acquire a “ground truth” dataset from rat cortex with 32- and 128-channel silicon polytrodes, which can now be used to validate methods for interpreting dense extracellular recordings and help resolve persistent debates about the nature and origin of the extracellular signal. This dataset, which will grow as new devices are fabricated, is available online (http://www.kampff-lab.org/validating-electrodes).

## MATERIALS AND METHODS

### Set-Up Design and Calibration

**Fig. 1.**
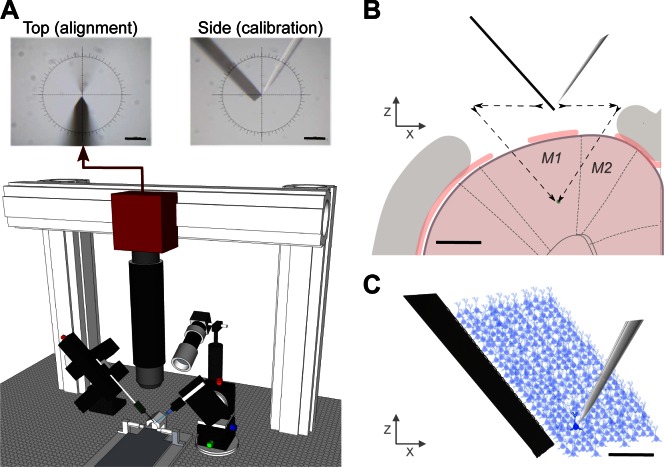
In vivo paired-recording setup: design and method. *A*: schematic of the dual-probe recording station. The “PatchStar” (PS) micromanipulator drives the juxtacellular pipette and the “In-Vivo Manipulator” (IVM) manipulator drives the extracellular polytrode. The setup includes a long working distance microscope assembled from optomechanical components mounted on a 3-axis motorized stage. The alignment image provides a high-resolution view from above the stereotactic frame, *top left*; however, a side-view can also be obtained for calibration purposes, *top right* (scale bar = 100 μm). *B*: schematic of a coronal view [reproduced from Paxinos G, Watson C. *The Rat Brain In Stereotaxic Coordinates* (6th ed.), Elsevier, 2007, with permission] of the craniotomy and durotomies with both probes positioned at the calibration point. The distance between durotomies, such that the probe tips meet at deep layers in cortex, was ∼2 mm. The black arrows represent the motion path for both electrodes entering the brain (scale bar = 1 mm). *C*: diagram of simultaneous extracellular and juxtacellular paired-recording of the same neuron at a distance of 90 μm between the micropipette tip and the closest electrode on the extracellular polytrode (scale bar = 100 μm).

The dual-recording setup requires two aligned, multi-axis micromanipulators (Scientifica) and a long working distance optical microscope (Fig. 1*A*). A “PatchStar” (PS) and an “In-Vivo Manipulator” (IVM) are mounted on opposite sides of a rodent stereotaxic frame. The stereotaxic frame also defined the common *X*-, *Y*-, and *Z*-axes to which the manipulators were aligned: *X*-axis is parallel to the medio-lateral axis; *Y*-axis is parallel to the anterior-posterior axis, and *Z*-axis is parallel to the dorso-ventral axis.

The probes were held at an angle: the PS allows the combination of two motion axes (*XZ*-axis in approach mode) and this approach angle was set at 61° from the horizontal, whereas the IVM, a rigid three-axis linear actuator, was tilted −48.2° from the horizontal around the *Y*-axis. The use of two different models of manipulator was a practical constraint in this study, as the IVM permitted a greater range of travel for initial prototyping. However, future dual-probe setups will utilize two PS systems and the calibration and operation procedures will remain identical.

#### Alignment microscope.

A custom microscope was assembled from optomechanic components (Thorlabs), a long-working distance objective (Infinity-Corrected Long Working Distance Objective, ×10; Mitutoyo), and a high-resolution CMOS camera (PointGrey). The numerical aperture of the objective (0.28) had a theoretical resolution limit of ∼1 μm in *X*- and *Y*-directions and ∼10 μm in *Z*-direction, which was oversampled by the camera sensor. Oblique illumination was necessary to acquire an image of both the extracellular probe and juxtacellular pipette, directly above the craniotomy, with sufficient contrast to accurately “zero” the position of each probe (Fig. 1*A*, *top left*). The repeatability of visually aligning each probe to the center of the image (“zeroing”) was evaluated by manually moving the tip of the pipette several times (*n* = 11) from outside the field-of-view to the focal plane and image center and recording the manipulator coordinates. The optical alignment procedure had 0.5 ± 0.5 μm repeatability in *XY* and 2.6 ± 1.7 μm in *Z*.

#### Mechanical alignment.

Ensuring that the axes from both manipulators are parallel began with a mechanical alignment procedure. The PS was “squared” with the stereotactic frame using a digital machinist's dial (Fine Reading Indicator; RS Pro) mounted in the electrode holder, using exactly the same procedure that a machinist uses to align a milling machine *XY*-table and -column. The dial was placed in contact with a planar surface of the stereotactic frame and moved along this surface (see Supplemental Material Movie S1; Supplemental Material for this article is available online at the Journal website). Any change in the micrometer-sensitive dial's readings during movement indicated a misalignment, and the manipulator was repeatedly “realigned” (i.e., tapped with a soft surface hammer) until this differential was minimized. The IVM manipulator was then aligned using the same procedure in the *Y*-axis using both the vertical and horizontal planes of the stereotactic frame.

#### Estimating misalignment.

Each probe tip was positioned, sequentially, at the center of the microscope image (indicated by an overlay crosshair) and the respective motorized manipulator coordinates were set to zero (*X* = 0, *Y* = 0, *Z* = 0; Fig. 1*A*). The pipette was then moved to a different position in space, the microscope was moved and refocused to recenter the tip of the pipette in the crosshair. Next, without moving the microscope, we moved the extracellular probe to the same coordinates as the pipette. If there were no misalignment between the two manipulators, then the probe should arrive at the center of the image crosshair. If the probe is not centered, then the amount of re-positioning required (in *X*, *Y*, and *Z*) to venter the probe provides an accurate measure of residual axis misalignment. These “errors” were recorded for each position as the probes were sequentially moved to several different locations (*n* = 15) that spanned a large volume (5,000 × 5,000 × 5,000 μm, in 1,000-μm steps). Following mechanical alignment, the average distance error recorded in this volume was 75.6 ± 36.2 μm (*n* = 15). Note that our manipulators were mounted with different approach angles (θ_IVM_ = 48.2°) and converting the coordinates of the IVM into the PS frame requires the following transformation and assume perfect *Y*-axis alignment.

$$X_{\text{PATCH}}=\cos\left(\theta_{\text{IVM}}\right)\cdot Z_{\text{IVM}}+\sin\left(\theta_{\text{IVM}}\right)\cdot X_{\text{IVM}}$$

$$Y_{\text{PATCH}}=Y_{\text{IVM}}$$

$$Z_{\text{PATCH}}=-\sin\left(\theta_{\text{IVM}}\right)\cdot Z_{\text{IVM}}+\cos\left(\theta_{\text{IVM}}\right)\cdot X_{\text{IVM}}$$

#### Software correction.

By using one manipulator as the reference, we can use the position errors measured at many different locations to estimate the coordinate transformation that best compensates for the misalignment of the second manipulator. We adopted the PS coordinate system as the reference frame and transformed the recorded IVM coordinates into the PS frame, in an affine manner, as follows for the *X*-axis: *X*_PATCH_ = *A*·*X*_IVM_, where *A* represents a transformation matrix that best matches these pairs of coordinates. The distance error estimated after the software alignment was reduced to 10.5 ± 5.2 μm (*n* = 15).

The protocol for the acquisition and transformation of axis position was implemented in Bonsai, an open-source visual programming framework, which can be freely downloaded at https://bitbucket.org/horizongir/bonsai (Lopes et al. 2015).

### Surgery

Rats (400–700 g, both sexes) of the Long-Evans strain were anesthetized with a mixture of ketamine (60 mg/kg ip) and medetomidine (0.5 mg/kg ip) and placed in a stereotaxic frame that was atop a vibration isolation table (Newport). Equipment for monitoring body temperature as well as a live video system for performing craniotomies and durotomies were integrated into the setup. At the initial stage of each surgery, atropine was given to suppress mucus secretion (atropine methyl nitrate; Sigma-Aldrich). Anesthetized rodents then underwent a surgical procedure to remove the skin and expose the skull above the targeted brain region. Small craniotomies (4 mm medial-lateral and 2 mm anterior-posterior) were performed above dorsal cortex. The craniotomy centers were 2.5 mm lateral to the midline and ranged from +4 to −4 anterior-posterior, thus exposing either motor, sensory, or parietal cortex. Two reference electrodes Ag-AgCl wires (Science Products E-255) were inserted at the posterior part of the skin incision on opposite sides of the skull.

### Dense Silicon Polytrodes

All experiments were performed with two different high-density silicon polytrodes. A commercially available 32-channel probe (A1x32-Poly3-5mm-25s-177-CM32; NeuroNexus), with 177-μm^2^ area electrodes (iridium) and an intersite pitch of 22–25 μm, was used in the first experiments. The impedance magnitude for these sites at 1 kHz was ∼1 MΩ, but for some experiments, a PEDOT coating was applied to lower this impedance to ∼50–100 kΩ. In later experiments, we used a 128-channel probe produced by the collaborative NeuroSeeker project (http://www.neuroseeker.eu/) and developed by IMEC using CMOS-compatible process technology. The probe's titanium nitride (TiN) electrodes were 400 μm^2^ (20 × 20 μm^2^) in size, were arranged at a pitch of 22.5 μm, and had an impedance magnitude of 50 kΩ at 1 kHz.

Before each surgery, the impedance magnitude of each electrode site was measured for diagnostic purposes using a protocol implemented by the amplifier/DAC chip (InTan Technologies). Following each surgery, cleaning was performed by immersing the probe in a trypsin solution [trypsin-EDTA (0.25%), phenol red; TermoFisher Scientific] for 30–120 min and rinsing with distilled water.

### Probe Insertion and Simultaneous Juxtacellular-Extracellular Recordings

After both the extracellular probe and juxtacellular pipette positions were sequentially “zeroed” to the center of the microscope image, the microscope was replaced by a macro-zoom lens (Edmund Optics) for visually guided insertion. The extracellular probe was inserted first, at a constant velocity of 1 μm/s, automatically controlled by the manipulator software. When the extracellular probe was in place the juxtacellular pipette filled with 1× PBS was then lowered through a second durotomy under visual guidance using the overhead surgery camera. We used capillary borosilicate glass tubing with flame polished ends, an outer diameter of 1.50 mm, inner diameter of 0.86 mm, and a length of 10 cm (Warner Instruments). The tubing was pulled into micropipettes using a laser-based micropipette puller (P-2000; Sutter Instruments). The resulting juxtacellular pipettes had resistances between 3 and 7 MΩ and tip diameter of about 1–4 μm. As the pipette approached the extracellular electrodes, we followed a protocol for performing loose-patch recordings from neurons as previously describe (Herfst et al. 2012). Positive pressure (25–30 mmHg) was reduced on the pipette to 1–10 mmHg (DPM1B Pneumatic Transducer Tester; Fluke Biomedical), and the amplifier for juxtacellular recordings (ELC-01X; NPI) was set to voltage-clamp mode (25-mV steps at 20 Hz). As the electrode was advanced towards a cell membrane, we observed an increase in the pipette resistance. If spikes were observed, the pressure was then released (0 mmHg) and a slight suction applied to obtain a stable attachment to the cell membrane.

A data acquisition board (National Instruments) was used to control amplifier voltage commands. However, after a stable recording was achieved, simultaneous recording of both extracellular and juxtacellular electrodes used exclusively the Open Ephys (http://www.open-ephys.org) acquisition board ADCs (for the juxtacellular signal) along with the RHD2000 series digital electrophysiology interface chip that amplifies and digitally multiplexes the extracellular electrodes (Intan Technologies). Extracellular signals in a frequency band of 0.1–7,500 Hz and juxtacellular signals in a frequency band of 300-8,000 Hz were sampled at 30 kHz with 16-bit resolution and were saved in a raw binary format for subsequent offline analysis using a Bonsai interface. For the analyses described in the following, a third order Butterworth filter with a band-pass of 100-14,250 Hz (95% of the Nyquist frequency) was used in the forward-backward mode. For some recordings we noticed a high-frequency noise contribution and we thus used a band-pass of 100-5,000 Hz.

All experiments were approved by the Champalimaud Foundation Bioethics Committee and the Portuguese National Authority for Animal Health, Direcção-Geral de Alimentação e Veterinária.

## RESULTS

### Setup Design

The “dual-probe” positioning and recording setup presented in Fig. 1*A* was designed to reliably target neural cell bodies located within ∼100 μm of the polytrode electrode sites without optical guidance. In this setup, the motorized manipulators, video capture, online visualization/control parameters, and extra- and juxtacellular voltage recording were integrated and coordinated by custom open-source software developed within the Bonsai framework (Lopes et al. 2015).

Following a mechanical alignment and software calibration of both manipulators' axes, each paired-recording experiment began with the optical “zeroing” of both probes. Each probe was positioned, sequentially, at the center of the microscope image (indicated by a crosshair) and the motorized manipulator coordinates set to zero (Fig. 1*A*). As shown in Fig. 1*B*, this alignment is performed directly above the desired rendez-vous point inside the brain, as close as possible above dura, usually between 1 and 4 mm but far enough to reduce background light reflected from the brain surface into the microscope image. During optical calibration it is possible to select any point on the extracellular electrode to be the origin (*X* = 0, *Y* = 0, *Z* = 0) by aligning that point of the probe in the reticle. However, the distance reported in the subsequent data is always the Euclidean distance between the tip of the pipette and the closest extracellular electrode.

With practice, multiple cells in the vicinity (<200 μm) of the polytrode could be recorded through multiple insertions of the juxtacellular pipette (Fig. 1*C* is a schematized example of one paired-recording).

Before the surgeries, we validated the alignment of the motors by moving both probes independently from the calibration point (0, 0, 0) towards a different point in space and recording the position difference between them after travel. During our experiments, the movement of the probes primarily occurred in the *XZ*-plane. We found that when we moved both probes to a new *Z*-position 3 mm below the calibration point (0, 0, −3), similar to an actual recording experiment (Fig. 1*B*), the distance error observed was 10.5 μm after the software calibration (and 31.6 μm before software calibration), which was acceptable for targeting the same region in cortex. During a recording, advancing the pipette very close to the extracellular probe surface (<30 μm) allowed direct detection of the 25-mV test pulse delivered by the juxtacellular amplifier (Supplemental Material Movie S2) on the extracellular array. The peak of this test pulse was largest on the targeted, and thus nearest, electrode site, providing further validation of our setup's positioning accuracy.

### Paired Juxtacellular-Extracellular Recording

Twenty-three neurons were recorded with a distance <200 μm between the juxtacellular pipette tip and the closest extracellular electrode within the cortex of anesthetized rats. The precision aligned dual-probe setup could efficiently target neurons nearby the extracellular probe, and for each insertion of the pipette at least one paired-recording was obtained. Eleven animals were used to record all the pairs in this study (the full dataset is summarized in Supplemental Material Table 1). However, with practice, it was possible to insert the juxtacellular pipette several times at different locations (max 4) and to record many neurons (max 6) along a single track.

**Fig. 2.**
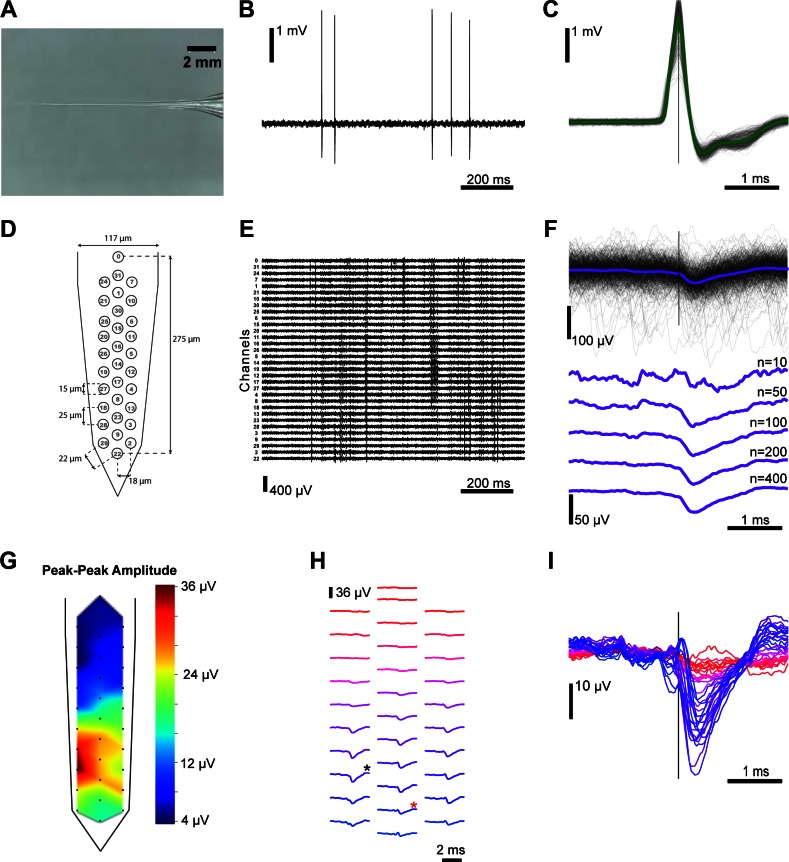
Paired extracellular and juxtacellular recordings from the same neuron. *A*: recording pipette with a long thin taper used for juxtacellular recordings with typical tip diameter of 1–4 μm and resistance of 3–7 MΩ. *B*: representative juxtacellular recording from a cell ∼1,256 μm in depth, 51 μm from the extracellular probe (*2014_10_17_Pair1.0*), with a firing rate of ∼ 1 Hz. *C*: juxtacellular action potentials are overlaid, time-locked to the maximum positive peak, with the average spike waveform superimposed (*n* = 442 spikes). *D*: extracellular dense polytrode array with a span of 275 μm along the shank axis; the electrode channel number is represented at each site. *E*: representative extracellular recording that corresponds to the same time window as the above juxtacellular recording. Traces are ordered from upper to lower electrodes and channel numbers are indicated. *F*: extracellular waveforms, aligned on the juxtacellular spike peak, for a single channel (channel 18) and the juxtacellular triggered average (JTA) obtained by including an increasing number of juxtacellular events (*n* as indicated). *G*: spatial distribution of the amplitude for each channel's extracellular JTA waveform. The peak-to-peak amplitude within a time window (±1 ms) surrounding the juxtacellular event was measured and the indicated color code was used to display and interpolate these amplitudes throughout the probe shaft. *H*: waveform averages for all the extracellular electrodes are spatially arranged. The channel with the highest peak-to-peak JTA amplitude (channel 18) is marked with a black asterisk and the closest channel (channel 9) is marked with a red asterisk. *I*: extracellular JTA time courses for each channel are overlaid and colored according to the scheme in *H*.

The juxtacellular pipette had a long thin taper to minimize tissue displacement during penetration and promote longer stable recordings (Herfst et al. 2012) (Fig. 2*A*). As the juxtacellular electrode was advanced through the brain, several neurons were encountered at different locations along the motion path and, consequently, at different distances from the extracellular polytrodes. Figure 2*B* illustrates the large juxtacellular (peak-to-peak ∼4 mV) signal recorded from a neuron encountered at a distance of 51.0 ± 10.5 μm between the micropipette tip and the closest extracellular electrode. The positive-before-negative biphasic waveform shape (Fig. 2*C*) is indicative of a capacitively coupled cell-attached recording from a somatic/perisomatic located recording pipette (Herfst et al. 2012). However, for two paired-recordings in the dataset, the pipette recording exhibited the waveform of well isolated extracellular spike (negative-before-positive), likely due to incomplete contact between the membrane and pipette presenting lower peak-to-peak amplitudes (*2015_09_04_Pair 5.0* and *2015_09_03_Pair 9.0*).

A simultaneous extracellular recording was made with the 32-channel probe illustrated in Fig. 2*D*, allowing us to specifically characterize the extracellular signature of an action potential generated by the juxtacellular recorded neuron. The band-pass filtered extracellular traces, ordered according to the electrode's geometry, are presented in Fig. 2*E* and correspond to the same time window as the juxtacellular recording (Fig. 2*B*). A short time window (4 ms) extracted from the extracellular trace around each detected juxtacellular event (occurrence of the action potential positive peak) for one extracellular channel is shown in Fig. 2*F*. Despite the low amplitude, a clear extracellular signature of the juxtacellular recorded neuron's spike can be recovered by averaging windows across multiple events. This juxtacellular triggered average (JTA) can be computed for all channels, allowing a high signal-to-noise estimate of the spatiotemporal distribution of the EAP. The JTA peak-to-peak amplitude for each channel interpolated within the electrode site geometry, sometimes called “the cell footprint” (Delgado Ruz and Schultz 2014), is shown in Fig. 2*G*. The JTA waveforms for each channel are shown, arranged using the relative probe spacing in Fig. 2*H* and overlaid in Fig. 2*I*.

The example presented in Fig. 2 is from one paired juxtacellular and extracellular recording. Several recordings were made in a similar manner and we next examined the variety of extracellular signatures obtained for different neurons at different positions relative to 32 and 128-channel dense polytrodes.

### Distance Dependence of Extracellular Signal Amplitude

**Fig. 3.**
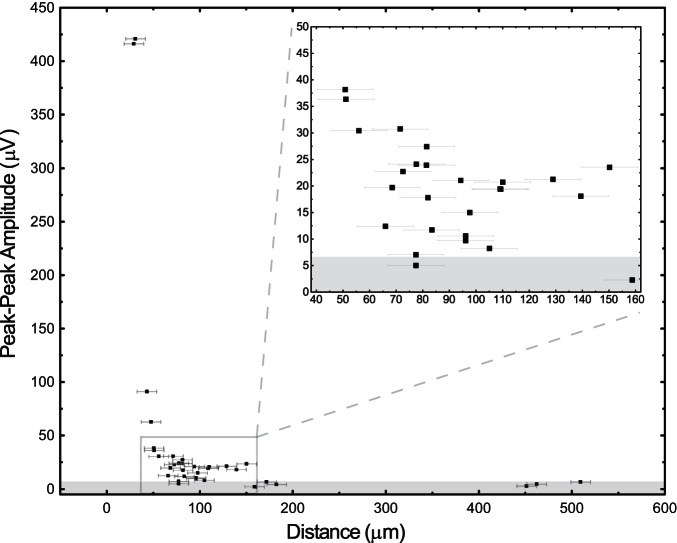
Distance dependence of extracellular signal amplitude. The maximum peak-to-peak amplitude of the JTAs (±1 ms of the alignment time) across all extracellular channels for each paired-recording vs. the distance between the closest extracellular electrode and the juxtacellular pipette tip. Horizontal error bars report uncertainty in position estimate (±10.5 μm). The gray shaded region indicates a 5-μV threshold for excluding possible cross-talk electrical artifacts between the extra- and juxtacellular recording electronics.

Following a stable juxtacellular recording we were sometimes able to move the extracellular probe and obtain another recording configuration/distance for the same neuron. The relationship between extracellular signal amplitude and distance from the probe for 35 such recording configurations, obtained from 23 neurons, is shown in Fig. 3 (we also included in the dataset 3 paired-recordings with distances >200 μm). Across all of our paired-recordings, the distance between a neuron and the extracellular electrodes was the major factor determining the peak-to-peak extracellular signal amplitude.

Large peak-to-peak amplitudes were only observed for neurons <50 μm from the nearest electrode, which is in accordance with previous measurements in hippocampus (Henze et al. 2000) and some theoretical models (Somogyvári et al. 2012). For neurons encountered within 50 to 150 μm of the probe surface, the magnitude of the neuron's extracellular signal ranged from 38 to 5 μV. All neurons encountered with a distance ≥150 μm did not show a canonical spike waveform, even after averaging, and we occasionally detected a small artifact (<5 μV at 0 ms) that was similar for all channels and likely due to cross talk between the extracellular and juxtacellular recording electronics. Nevertheless, we include these distant cells in the dataset since they could potentially be used to better understand the spike-local field potential relationship (Lewis et al. 2015; Berényi et al. 2014).

### Detection of the Juxtacellular Spikes on the Extracellular Probe

The first step in the analysis of extracellular data is the identification of discrete spike events (Hazan et al. 2006). Therefore, to use paired recordings to evaluate algorithms for assigning these extracellular events to clusters belonging to distinct neurons (i.e., spike sorting), one must be able to detect the juxtacellular spike on the extracellular electrodes. We used a popular spike detection algorithm, SpikeDetekt, which extracts action potentials as spatiotemporally localized events (Rossant et al. 2016), to identify all spikes visible to our extracellular probe. SpikeDetekt uses a high threshold to detect spikes on a single channel and then a lower threshold to associate neighboring channels (using a flood-fill algorithm) that sense the same spike. We used the same detection parameters for our entire dataset: third-order Butterworth, forward-backward mode, band-pass filter (500-14,250 Hz) and strong and weak threshold levels of 4.5 and 2 times the standard deviation, respectively. The juxtacellular spike times were determined as the peaks of well-isolated threshold crossings (see Supplemental Material Table S1 for the threshold values used for each individual paired-recording).

**Fig. 4.**
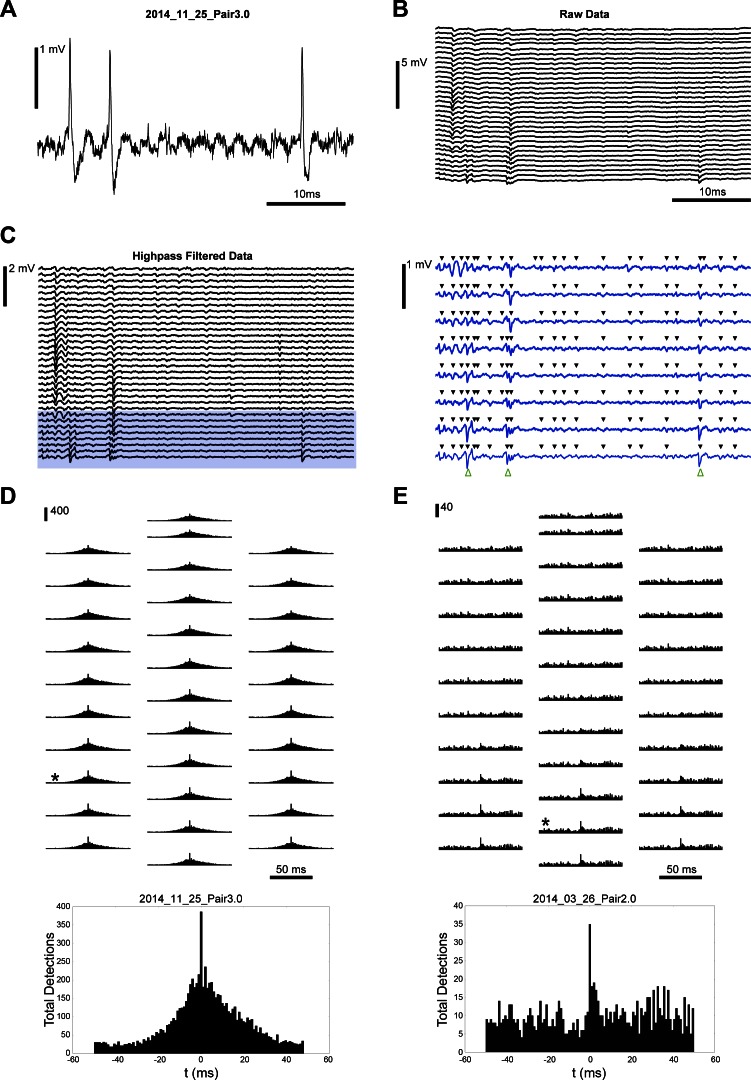
Extracellular detection of the juxtacellular neuron's action potentials. Representative juxtacellular recording (*A*) and wide-band (*B*; 0.1–7,500 kHz) signal recorded simultaneously with a 32-channel silicon polytrode. *C*: on the highpass filtered extracellular data is visible the occurrence of temporally overlapping spikes on separated electrodes. The highlighted traces are expanded in the right panel and included black arrows to indicate all spikes identified by SpikeDetekt using standard thresholds and green arrows to indicate the time of juxtacellular spikes. *D*: peri-event time histograms of the extracellular spike events found by SpikeDetekt, relative to the juxtacellular spike times in 1-ms bins centered at 0 ms, are shown for each electrode channel at their relative position on the extracellular probe. The channel with the largest peak in the bin at 0 (±0.5 ms from the juxtacellular event) is indicated by asterisk and expanded at *bottom*. *E*: same presentation as in *D* but for a neuron with a smaller extracellular action potential.

This spike detection process is illustrated in Fig. 4, *A–C*, for a data segment containing the contribution of a neuron that was simultaneously recorded with the juxtacellular pipette (*2014_11_25_Pair3.0*). To compare the extracellularly detected event times with the spike times observed in the juxtacellular recording, we generated a peri-event time histogram (PETH) using all spike events found by SpikeDetekt on the extracellular channels aligned relative to each juxtacellular spike (Fig. 4, *D* and *E*). In some paired-recordings, these PETHs reveal a high probability of spike co-occurrence at 0 ms, indicating that the juxtacellular neuron's spike is being found by SpikeDetekt. The count value in the PETH 0-ms bin for the recorded pair in Fig. 4*D* (*2014_11_25_Pair3.0, channel 18*) suggests that all of the juxtacellular spikes were found but that this bin also includes detections of coincident spiking events occurring in the background neural activity (386 detected, 348 actual). These false positives could potentially be distinguished through sorting. In contrast, the PETH 0-ms bin for the pair in Fig. 4*E* (*2014_03_26_Pair2.0, channel 9*) indicates that only 23% of the total number of juxtacellular spikes were detected (35 detected, 150 actual), some of which are likely to also reflect coincident background events. We note that a larger number of putative juxta spikes could be recovered in this recording by reducing the high threshold level used by SpikeDetekt but this would also include more background events and likely complicate subsequent sorting. The two examples in Fig. 4 thus highlight both an “easy” and “hard” case for spike detection algorithms, which must be further developed in coordination with sorting algorithms, to better utilize the rich information provided by dense polytrodes.

### Spatiotemporal Structure of Extracellular Signatures

**Fig. 5.**
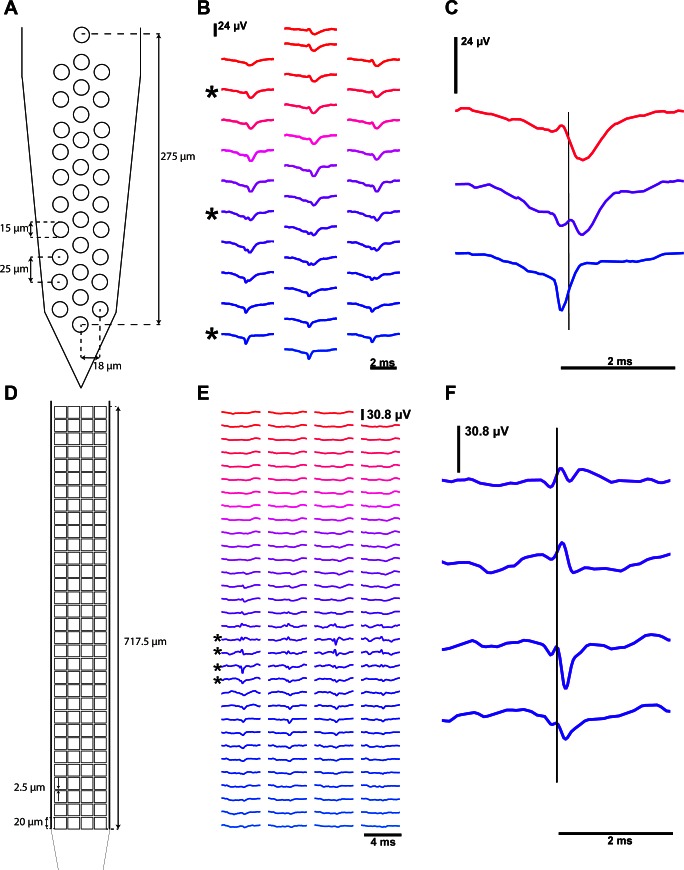
Spatiotemporal structure of extracellular signatures. *A*: geometry and dimensions of the 32-channel electrode array. *B*: JTA waveforms (*2014_11_25_Pair1.1*) for all the extracellular electrodes are spatially arranged according to the probe geometry. *C*: expanded comparison of the JTA waveforms for the indicated electrodes with a line denoting the peak time of the juxtacellular spike. *D–F*: similar presentation as (*A–C*) for one 128-channel polytrode pair example (*2015_09_04_Pair5.0*).

**Fig. 6.**
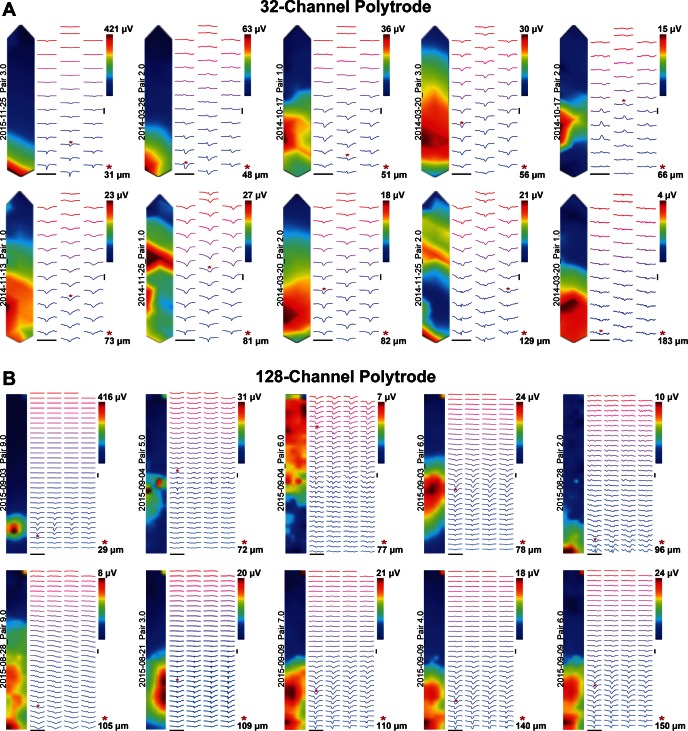
Dataset for validating spike detection and sorting algorithms for dense polytrodes. *A*: spatial distribution of the peak-to-peak amplitude within a time window (±1 ms) surrounding the juxtacellular event and the indicated color code was used to display and interpolate these amplitudes throughout the 32-channel probe shaft. In addition, the extracellular JTA waveforms for all the extracellular electrodes are spatially arranged. *B*: same presentation as in *A* for paired-recordings with the 128-channel probe.

Neurons near the polytrode surface exhibited a rich diversity of action potential waveforms (amplitude and dynamics) spread across multiple electrode sites (Fig. 6 and Supplemental Material Movies S3 and S4). This spatiotemporal structure will not only provide additional information for improving spike detection and sorting procedures, but may also reveal specific contributions from different parts of the neural membrane to this extracellular signature. For example, in Fig. 5*C*, the first negative peak (blue trace) in the extracellular potential is hypothesized to arise from currents in the distal axon initial segment (Shu et al. 2007; Hu et al. 2009) and the later peaks (purple and red traces) might then be due to the backpropagation to soma and dendrites (Buzsáki et al. 1998). The propagation velocity, estimated from the distance of the recording sites and the delay between the negative peaks (blue and red traces in Fig. 5*C*) was ∼0.55 m/s, which is in agreement with the value found in the literature for backpropagation of action potentials in cortical pyramidal cells 0.67 ± 0.11 m/s (Buzsáki et al. 1998). Another example of complex structure in the extracellular signature is seen in Fig. 5*E*, in this case the primary signal is localized to a small region of electrodes and varies greatly between neighboring sites, which are separated by only 2.5 μm. These examples, and others in our dataset, clearly suggest that the amount of useful spatiotemporal information captured by dense large-scale neural recording devices is promising (Buzsáki 2004), not only for improving algorithms that detect and sort events but also to identify cell types based on the morphology suggested by their extracellular signature.

## DISCUSSION

In the present study, our dual-recording setup allowed precise targeting of both an extracellular probe and a juxtacellular pipette to the same position in cortex. The setup is low cost and easily implemented by any electrophysiology laboratory with two motorized (servo/stepper) micromanipulators. We hope that our description will instigate the collection of such critical cross-validation data from the forthcoming deluge of novel neural recording devices.

### Dataset for Cross-Validating Polytrodes and Spike Detection/Sorting Algorithms

A summary of the current cross-validation dataset is presented in Fig. 6. It includes twenty juxta-extracellular pairs recorded with both 32- and 128-channel polytrodes, at a range of interprobe distances and depths in cortex (800 to 1,800 μm from the pial surface). Future experiments, which use cell-attached labeling to anatomically reconstruct the juxtacellular neuron following a paired-recording, are now being pursued to extend this validation dataset. However, the existing dataset already includes a number useful cross-validation examples: nearby cells with large EAPs, which will provide “ground truth” data for evaluating current spike detection and sorting algorithms, as well as more challenging intermediate cells, for which new algorithms, specifically optimized to use the additional information available to dense silicon polytrodes, may be able to recover. The full dataset, as well as probe maps and analysis code, is available online (http://www.kampff-lab.org/validating-electrodes/) and summarized in Supplemental Material Table S1.

### Are We Missing Neurons with Extracellular Recording?

An apparent discrepancy in the number of neurons that are reported to be active with optical (∼50%) vs. electrical (<10%) recording techniques has prompted a number of researches to ask whether extracellular recording is “missing something” (Buzsáki 2004; Shoham et al. 2006). Many explanations have been proposed (extracellular recording and sorting methods are biased to highly active neurons, some neuron types have weak or more localized extracellular signatures, etc.), yet estimating how many neurons an electrode should detect depends critically on knowing the volume of neural tissue to which an electrode is sensitive (i.e., from how far away can a neuron's spike be detected?).

The literature is rather inconsistent, but there a number of reports of recording neurons extracellularly (>50 μV) from >100 μm away (Henze and Buzsáki 2007; Du et al. 2011), while others suggest, based on modeling (Gray et al. 1995; Shoham et al. 2006; Somogyvári et al. 2012; Delgado Ruz and Schultz 2014) and “ground truth” measurements (Henze et al. 2000; Anastassiou et al. 2015), that the limit is in fact closer to ∼50 μm. Our data are consistent with the latter estimates (the maximum distance at which we observed a large peak-to-peak amplitude spike was only 48 μm) but also suggest a possible explanation for these discrepant views.

We propose that when an extracellular probe insertion is aligned with the axis of a pyramidal neuron's apical dendrite (or any neuron with an elongated morphology), then its EAP will be visible over a large distance, roughly matching the extent that the cell's dendritic arbor passes nearby the probe (Buzsáki et al. 1998). However, as the probe is positioned further away laterally from the neuron soma (perpendicular to the major axis of the neuron) then the EAP amplitude falls off steeply. All of our recordings used an extracellular array that was inserted parallel to the apical dendrites of cortical pyramidal neurons (Fig. 1*B*) and perpendicular to the cortical laminae. Therefore, if we were juxtacellularly recording from a pyramidal neuron whose soma was nearby the electrode surface and whose apical dendrite was aligned with the probe surface, then we would expect to detect a strong EAP across a large portion of the electrode surface, albeit with varying temporal waveforms (Figs. 5*B* and 6). However, if we juxtacellularly record from neurons whose soma are further from the probe surface, the size of the EAP on the probe surface will decrease rapidly (Fig. 3). This interpretation can explain why one neuron might occasionally be detected over hundreds of micrometers (i.e., that neuron's morphology happened to be aligned with the probe/insertion track), but still supports the conclusion that an extracellular electrode is primarily sensitive to neurons (and their processes) within a 50 μm radius. Given this limited sensitivity range, are we still missing neurons? Based on cellular density estimates for cortex (40,000 to 60,000 neurons/mm^3^; DeFelipe et al. 2003), and the half-spherical volume in front of a polytrode electrode, then we would expect each site to be sensitive to ∼10–15 neurons. These estimates are consistent with reported results for dense silicon polytrodes in cortex (Blanche et al. 2005). Our data thus suggest that there may not be a “dark neuron” problem but rather that extracellular electrodes are sensitive to a much smaller volume than is sometimes proposed. However, much more data, from different brain regions containing diverse cell types, will be required to resolve this critical issue. We propose that our new dual-recording method will make gathering such important validation data much, much easier.

## GRANTS

This work was supported by funding from the European Union's Seventh Framework Programme (FP7/2007–2013) Grant Agreement 600925 and the FCT-MCTES Doctoral Grant SFRH/BD/76004/2011 (to J. P. Neto) and Bial Foundation Grant 190/12. Institutional support and funding was provided by the Champalimaud Foundation and Sainsbury Wellcome Centre (funded by the Gatsby Charitable Foundation and the Wellcome Trust).

## DISCLOSURES

No conflicts of interest, financial or otherwise, are declared by the author(s).

## ENDNOTE

At the request of the authors, readers are herein alerted to the fact that additional materials related to this manuscript may be found at the institutional website of one of the authors, which at the time of publication they indicate is: http://www.kampff-lab.org/validating-electrodes. These materials are not a part of this manuscript, and have not undergone peer review by the American Physiological Society (APS). APS and the journal editors take no responsibility for these materials, for the website address, or for any links to or from it.

## AUTHOR CONTRIBUTIONS

J.P.N., G.L., J.F., and A.R.K. conception and design of research; J.P.N., J.F., J.N., and P. Baião performed experiments; J.P.N., J.F., P.L., and A.R.K. analyzed data; J.P.N., J.F., P.L., and A.R.K. interpreted results of experiments; J.P.N., J.F., and A.R.K. prepared figures; J.P.N. and A.R.K. drafted manuscript; J.P.N., G.L., J.F., P.L., A. Aarts, A. Andrei, S.M., E.F., P. Barquinha, and A.R.K. edited and revised manuscript; J.P.N. and A.R.K. approved final version of manuscript.

## Supplementary Material

Movie 1

Movie 2

Movie 3

Movie 4

Table 1
